# Climate oscillation and the invasion of alien species influence the oceanic distribution of seabirds

**DOI:** 10.1002/ece3.6621

**Published:** 2020-08-20

**Authors:** Julian Perez‐Correa, Peter Carr, Jessica J. Meeuwig, Heather J. Koldewey, Tom B. Letessier

**Affiliations:** ^1^ Zoological Society of London Institute of Zoology London UK; ^2^ Escuela de Ciencias Ambientales Facultad de Ingeniería Universidad Espíritu Santo Samborondón Ecuador; ^3^ Imperial College London London UK; ^4^ Centre for Ecology and Conservation University of Exeter Cornwall UK; ^5^ Centre for Marine Futures, Oceans Institute and School of Animal Biology The University of Western Australia Crawley WA Australia; ^6^ Conservation and Policy Zoological Society of London London UK

**Keywords:** boosted regression tree, British Indian Ocean Territory, Chagos Archipelago, island invasive species, marine protected areas, *Rattus rattus*, ship rat

## Abstract

Spatial and temporal distribution of seabird transiting and foraging at sea is an important consideration for marine conservation planning. Using at‐sea observations of seabirds (*n* = 317), collected during the breeding season from 2012 to 2016, we built boosted regression tree (BRT) models to identify relationships between numerically dominant seabird species (red‐footed booby, brown noddy, white tern, and wedge‐tailed shearwater), geomorphology, oceanographic variability, and climate oscillation in the Chagos Archipelago. We documented positive relationships between red‐footed booby and wedge‐tailed shearwater abundance with the strength in the Indian Ocean Dipole, as represented by the Dipole Mode Index (6.7% and 23.7% contribution, respectively). The abundance of red‐footed boobies, brown noddies, and white terns declined abruptly with greater distance to island (17.6%, 34.1%, and 41.1% contribution, respectively). We further quantified the effects of proximity to rat‐free and rat‐invaded islands on seabird distribution at sea and identified breaking point distribution thresholds. We detected areas of increased abundance at sea and habitat use‐age under a scenario where rats are eradicated from invaded nearby islands and recolonized by seabirds. Following rat eradication, abundance at sea of red‐footed booby, brown noddy, and white terns increased by 14%, 17%, and 3%, respectively, with no important increase detected for shearwaters. Our results have implication for seabird conservation and island restoration. Climate oscillations may cause shifts in seabird distribution, possibly through changes in regional productivity and prey distribution. Invasive species eradications and subsequent island recolonization can lead to greater access for seabirds to areas at sea, due to increased foraging or transiting through, potentially leading to distribution gains and increased competition. Our approach predicting distribution after successful eradications enables anticipatory threat mitigation in these areas, minimizing competition between colonies and thereby maximizing the risk of success and the conservation impact of eradication programs.

## Graphical abstract

Climate oscillations may cause shifts in seabird distribution, possibly through changes in regional productivity and prey distribution. Invasive species eradication and subsequent island recolonization can lead to predictable distribution gains and increased competition. Our analysis predicting range extension after successful eradications enables anticipatory threat mitigation in these areas, minimizing competition between colonies, and thereby maximizing the risk of success and the conservation impact of eradication programs.

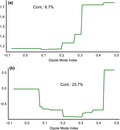

## 1 INTRODUCTION

Seabirds are apex predators in marine ecosystems (Ballance, Pitman, & Fiedler, 2006; Estes, Crooks, & Holt, 2001) and play important roles in ecosystem functionality (Fukami et al., 2006; Graham et al., 2018; Loder, Ganning, & Love, 1996; Schmidt, Dennison, Moss, & Stewart, 2004). Many seabirds, such as boobies (Sulidae), shearwaters, and petrels (Procellariidae), complete their life cycle primarily at sea and feed predominantly on pelagic forage species (e.g., anchovies, flying fish, euphausiids; Brooke, 2004; Votier & Sherley, 2017). Multiple threats to seabirds occur throughout their distribution, which is impacted by climatic variability and invasive species (Croxall et al., 2012; Dias et al., 2019). A recent assessment revealed that 69.7% of global seabird populations, including the families Laridae, Sulidae, and Procellariidae, are declining (Paleczny, Hammill, Karpouzi, & Pauly, 2015).

By linking terrestrial and oceanic trophic webs, seabirds can be sensitive indicators of long‐term and large‐scale changes in both environmental conditions and human activities (Einoder, 2009; Piatt, Sydeman, & Wiese, 2007). At sea, seabird distribution is closely linked to that of their prey. Oceanographic conditions (e.g., sea surface temperature, chlorophyll a) and geomorphic characteristics (e.g., slope, depth) are therefore commonly used as a proxy of food availability (Fox et al., 2017; Hyrenbach, Veit, Weimerskirch, Metzl, & Hunt, 2007; Mannocci, Catalogna, et al., 2014; Mannocci, Laran, et al., 2014; Maxwell & Morgan, 2013; Vilchis, Ballance, & Fiedler, 2006).

In the Indian Ocean (IO), several studies have explored linkages between oceanographic and geomorphic conditions and seabird distribution derived from single‐year surveys (e.g., Kappes, Weimerskirch, Pinaud, & Le Corre, 2011; Mendez et al., 2017; Weimerskirch, Le Corre, Jaquemet, & Marsac, 2005). Hyrenbach et al. (2007) explored drivers of seabird distribution in the southern IO, identifying the influence of sea surface temperature (SST) and proximity to sub‐Antarctic Islands. Mannocci, Laran, et al. (2014) found that their distribution in the southwest IO was closely related to persistent oceanographic conditions and that time‐averaged values over the long term (>7 years) were more predictive of distribution than those averaged over the short‐term (1 week).

Multiyear studies can provide managers with useful information needed to anticipate how interannual climate oscillations such as El Niño Southern Oscillation (ENSO; Sprogis, Christiansen, Wandres, & Bejder, 2018) and the IO Dipole (Saji, Goswami, Vinayachandran, & Yamagata, 1999) may impact seabird distribution. Interannual and climate variability in the tropical IO is to a large degree characterized by oscillations in SST gradient between the eastern and western basin, referred to as the IO Dipole (Saji et al., 1999). This gradient is represented by the Dipole Mode Index (DMI), where positive values correspond to cooler waters in the eastern basin and warmer in the west, whereas negative values correspond to warmer waters in eastern basin and colder in the west. Although the influence of climate oscillations and seabird dynamics has been the subject of a vast body of work (reviewed in Oro, 2014), the effects of the dipole on higher trophic levels remain poorly understood. A few studies have explored linkages between the IO Dipole and seabird population dynamics and behavior (Rivaland, Barbraud, Inchausti, & Weimerskirch, 2010; Tryjanowski, Stenseth, & Matysioková, 2013), due in part to its relatively recent discovery (Ashok, Guan, & Yamagata, 2003). Recent research on land birds has shown a positive correlation between the IO Dipole and bird community composition (Mehta & Wilby, 2018). However, to our knowledge, the influence of climate variability on seabird distribution in the central Indian Ocean has not been studied.

While seabird distribution at sea may fluctuate as a function of climatic variability, such as that reflected by the IO Dipole (Dias et al., 2019), distribution at sea is likely also impacted by invasive species on nearby islands. Island invasion by rodents, such as the ship rat *Rattus rattus*, is one of the greatest threats to seabird populations (Dias et al., 2019; Jones et al., 2008; King, 1985). Seabirds are central place foragers and thus sensitivity to rats may restrict their distributions in the water adjacent to invaded islands. Seabirds require islands to rest and breed, and both survival and breeding success rates are highest on islands with limited disturbance (King, 1985). Rats successfully invade islands by quickly adapting to new habitats, in part because of their omnivorous diet. Rats prey on both chicks and adults, causing population declines which can lead to extirpation (Fleet, 1972; Jones et al., 2008; King, 1985; Major, Jones, Charette, & Diamond, 2007). The impact of rat invasion on seabirds is species‐dependent and depends upon a combination of biological traits, such as breeding strategy, body weight, and life history. For example, small seabirds nesting in burrows such as storm‐petrels are particularly vulnerable to rat predation (Jones et al., 2008; Woodward, 1972). Rat‐eradication programs are considered a major component of successful island restoration and seabird population recovery (Borrelle, Boersch‐Supan, Gaskin, & Towns, 2018; Hutton, Parkes, & Sinclair, 2007; Le Corre et al., 2012; Russell & Holmes, 2015; Towns et al., 2009).

The IO has 27 archipelagos that are considered hotspots of marine biodiversity (Carr et al., 2020, Danckwerts et al., 2014; Le Corre et al., 2012), most of which are particularly important to seabirds (Le Corre & Jaquemet, 2005). In the central IO, the Chagos Archipelago, encompassed within the British Indian Ocean Territory (BIOT), is comprised of 55 tropical islands and was designated a no‐take marine protected area (MPA) in 2010. The majority of the archipelago has been closed to human activities since 1971 and is therefore relatively undisturbed (Everaarts et al., 1999; Readman et al., 2013; Sheppard & Sheppard, 2019). The archipelago is considered of great importance for seabird conservation, harboring eighteen species of resident breeders, and ten designated and two proposed “Important Birds Areas” (Hilton & Cuthbert, 2010; McGowan, Broderick, & Godley, 2008). Activities surrounding the historical coconut plantations, dating back to the turn of the 18th century, led to invasions of ship rats and other invasive mammals (i.e., feral cats *Felis catus*; Wenban‐Smith & Carter, 2016) on 26 islands (95.3% of the island area), negatively affecting the seabird populations (Harper & Bunbury, 2015; Harper, Carr, & Pitman, 2019; Hilton & Cuthbert, 2010). Seabird densities on rat‐free islands are up to 760 times greater than that on invaded islands, leading to nutrient subsidies and increased productivity on adjacent coral reefs (Graham et al., 2018). Notably, these subsidized reefs may recover faster following coral bleaching (Benkwitt, Wilson, & Graham, 2019), primarily enhanced by biodiversity richness and ecosystem functionality (Benkwitt, Wilson, & Graham, 2020). As productivity near rat‐free islands is enriched, it is therefore conceivable that seabirds on these islands have greater opportunities to feed in proximity to their colonies. After the successful eradication of rats from Île Vache Marine in 2017 (Harper et al., 2019), further rat eradication has been designated a priority target within the conservation framework of the BIOT Draft Conservation Management Plan 2018 – 2023 (BIOT Administration, 2018).

Here, we expand on previous work done in the IO (i.e., Hyrenbach et al., 2007; Mannocci, Laran, et al., 2014) by using a multiyear seabird survey (from 2012 to 2016) within the BIOT MPA to identify drivers of distribution. First, we modeled seabird distribution using oceanographic variables, and distance to the nearest island, in order to make general seabird distribution predictions and to establish the influence of oceanography and interannual variability. Then, having established that the at‐sea distribution of these species is in fact sensitive to the nearest island, we built new models considering distance to closest rat‐free island or to closest rat‐invaded island. Finally, predictions based on the rat‐invaded island model were subtracted from those of the rat‐free model to infer the spatial effect of rats on seabird distribution at sea during transiting or foraging. This approach enables us to estimate potential suitable marine habitats (i.e., distribution gain) in a scenario of a successful archipelago‐wide rat‐eradication program and possible factors relevant to island restoration priorities.

## 2 METHODS

### 2.1 Study area

The Chagos Archipelago is located in the central IO at 6°S and 72°E at the southern limit of the Chagos–Laccadive ridge and is over 1,500 km from the nearest continental land mass (Carr, 2012). Fifty‐five islands are clustered within the atolls of Diego Garcia, Peros Banhos, Salomon, Egmont, and on the Great Chagos Bank (Figure 1a) and constitute combined approximately 60 km^2^ of land area. The territory encompasses approximately 60,000 km^2^ of shallow photic reefs and 580,000 km^2^ of primarily oceanic habitat, with a maximum depth over 6,000 m (Carr, 2011; Dumbraveanu & Sheppard, 1999). The climate is tropical, characterized by oceanic conditions and the seasonal reversal monsoon (Sheppard, 1999). Situated in the intertropical convergence zone (ITCZ), the archipelago has moderate winds generally from the northwest (October to April) and the southeast (May to September). Sea surface temperature has an approximately bimodal distribution with maxima in December–January and March–April with a yearly mean of 28°C (Pfeiffer, Dullo, Zinke, & Garbe‐Schönberg, 2008) oscillating between 24.8 and 30.5°C.

**FIGURE 1:**
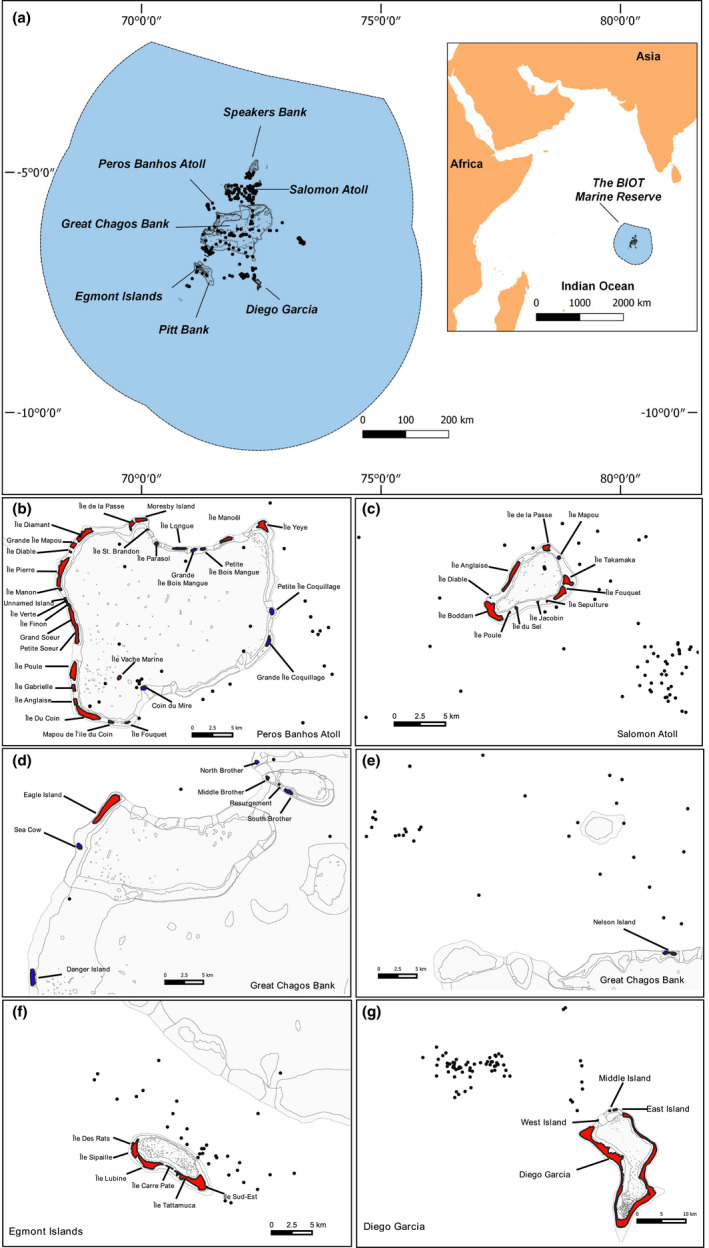
Seabird sampling effort within the Chagos Archipelago and the British Indian Ocean Territory. Boundaries of the BIOT MPA (a) and locations within the Indian Ocean (inset). Individual atolls and islands (b–g). Black dots represent the location of individual bird transects. Rat‐invaded islands are colored in red and rat‐free islands in blue. Six islands have been reported with uncertain rat status (Île Verte, Île Manon, Île Finon, Île de la Passe, and a small Unnamed Island in Peros Banhos) and are for representation purposes shown as rat‐invaded

### 2.2 Seabird observations

In order to identify the influence of oceanographic conditions and island on seabird distribution, we conducted a multiyear survey of the archipelago's seabirds at sea. The survey ran from 2012 to 2016 between November and April, to overlap with the moderate phase of the monsoon. This period generally coincides with peak seabird breeding activity in the Chagos Archipelago (Carr, 2011, 2015; Carr et al., 2020). During the months of sampling, the BIOT MPA and the IO experienced two seasons of modestly positive IO Dipole (2012–2013), which was followed by three neutral IO Dipole events (2014–2016; NOAA ESRL Physical Sciences Laboratory [NOAA ESRL] 2020).

Seabird transects (*n* = 317) were conducted from the BIOT patrol vessel. Our sampling was primarily designed to target focal sites that were typical pelagic habitats within the MPA, such as shallow seamounts (<70 m), deep banks (ca. 400 m), deep seamounts (<900 m), and deep basins (<2,000 m). Transects were generated by adapting the method of Tasker, Jones, Dixon, and Blake (1984). Each transect had a duration of 30 min, during which the vessel typically steamed at 12 knots and travelled ca. 11 km. All transects were generated within a 180° arc forward of the ship, out to approximately 300 m (Table 1, Figure 1). Each year during the survey, sampling effort was a trade‐off between partial replacement of the previous years to ensure a time series, and the addition of new locations, to increase the diversity of habitats surveyed. This meant that in certain years, sampling was more evenly spread throughout the archipelago (2012 and 2016), and more clustered in others (2013, 2014, 2015, Figure 2).

**TABLE 1:** Number of transects made by month and year within the BIOT MPA

Sampling Month	Sampling Month	Transects
2012	November 2012	50
2012	December 2012	40
2013	February 2013	7
2013	March 2013	3
2014	March 2014	10
2014	April 2014	5
2015	January 2015	92
2015	March 2015	1
2016	February 2016	109

**FIGURE 2:**
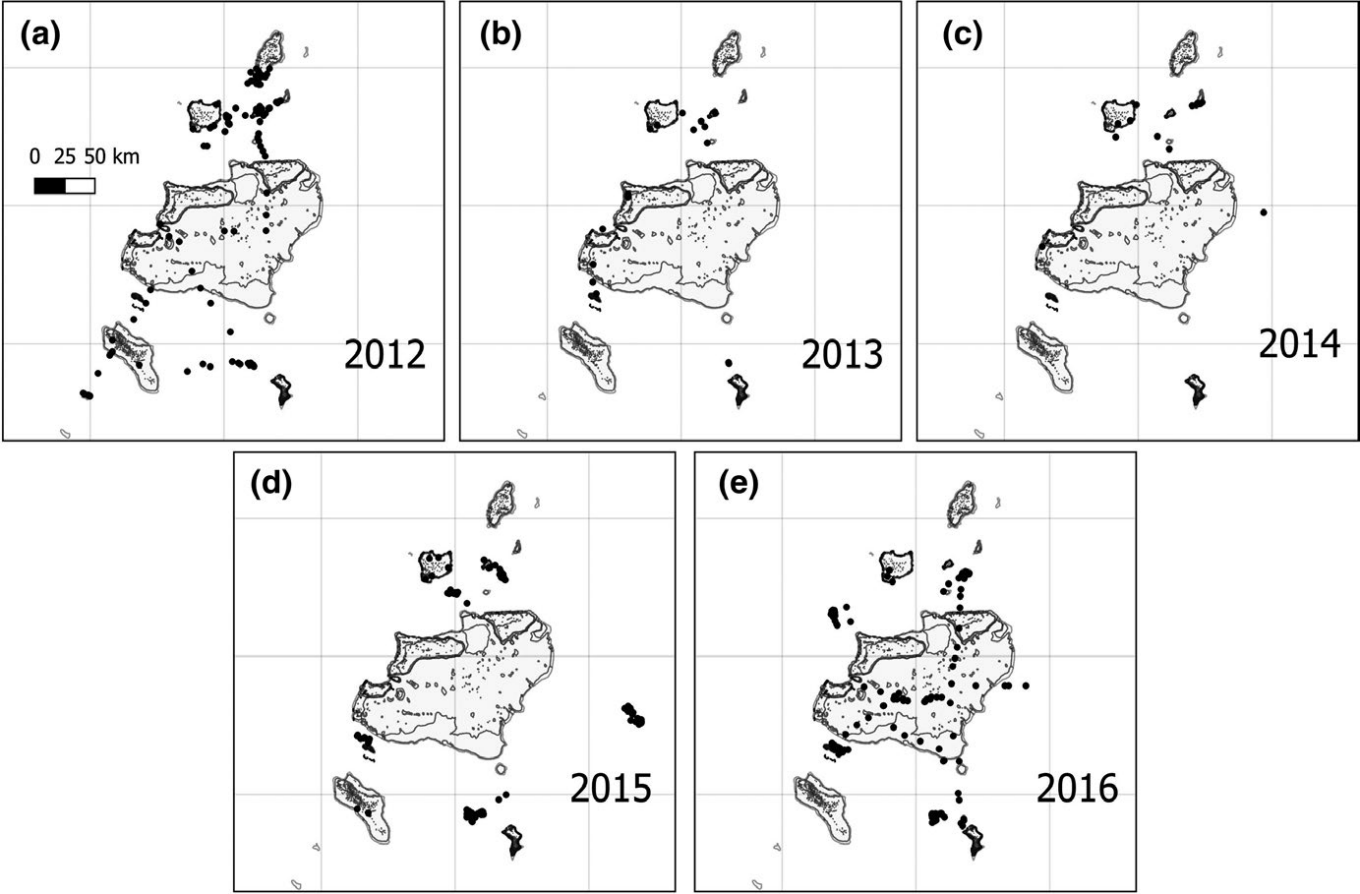
Year‐to‐year sampling effort in the Chagos Archipelago. Each 300‐m transect is represented by a black dot as the spatial resolution does not allowed us to perfectly draw lines

All seabird observations were led by Pete Carr, an expert on seabirds within the archipelago (e.g., Carr, 2011; Carr, 2012; Carr, 2015), supported by 1–2 field assistants. This consistency in the same lead observer with multiple field assistants and the use of transects with limited strip (i.e., 300 m) reduced potential sources of bias (Spear, Ainley, Hardesty, Howell, & Webb, 2004).

### 2.3 Oceanic habitat modeling

#### 2.3.1 Variables selection

We retained the most frequent and abundant seabird species (total sum of observations > 100) in the BIOT MPA in order to model oceanic distributions. These distributions were modeled based on geomorphic and oceanographic variables using Boosted Regression Tree models (BRT), an advanced form of regression (Friedman, Hastie, & Tibshirani, 2000) that use boosting to combine and adapt large numbers of relatively simple tree models, enabling model performance optimization (Elith, Leathwick, & Hastie, 2008).

The BRTs were fitted using individual species count per sample (a proxy for abundance) as the response variables, against explanatory variables that previously have been shown to contribute to seabird distribution (Fox et al., 2017; Mannocci, Catalogna, et al., 2014; Mannocci, Laran, et al., 2014; Vilchis et al., 2006; Yen, Sydeman, & Hyrenbach, 2004). Slope and distance to nearest island were calculated using QGIS version 3.8. Slope was derived from seabed depth values from a GEBCO 30‐arc seconds bathymetry grid (Becker et al., 2009), whereas distance to coast was obtained in function to the nearest island from each transect. Oceanographic variables were sea surface temperature (SST), chlorophyll a (CHL) concentration, and sea‐level anomaly (SLA). SST and CHL were obtained from aqua‐MODIS sensor at 4 km spatial resolution from Ocean Color Web (NASA Ocean Biology Processing Group, 2015). The SLA values were obtained from E.U. Copernicus Marine Service Information (2018). For each oceanographic covariate, we used the long‐term average for the month of sampling, computed over the last 15 years (2002–2017) following recommendations by Mannocci et al. (2017) for modeling mesoscale distributions of highly mobile animals, such as seabirds. Time‐averaged oceanographic variables have been shown to be more predictive of apex predators than short‐term values (Mannocci et al., 2017; Suryan, Santora, & Sydeman, 2012) and are more directly related to habitat consistency and thus ecologically relevant (Mannocci, Laran, et al., 2014). We also included an index of climatic variability, as represented by the Dipole Mode Index [DMI] and oceanic Niño index [ONI]. ONI and DMI were downloaded from NOAA (2020, 2020b) repository.

#### 2.3.2 Species distribution models

We first constructed full BRT models that included all explanatory variables (seven variables) for each species. Secondly, we observed the individual contribution of each variable and rebuilt the models selecting the variables with contributions greater than 5%. We then used the total explained deviance (TED) to evaluate the explanatory power of the models. TED was calculated dividing the residual deviance by the null deviance resulting from each selected species models. BRT models were fitted using the package *gbm* on R (R Development Core Team 2018 version R version 3.5.2) with code modifications provided by Elith et al. (2008). As recommended by Elith et al. (2008) and D'agata et al. (2014), we did an analytical exploration of BRT models in order to find a “trade‐off” between numbers of trees (nt; number of interactions), learning rate (lr; the shrinkage parameter), and tree complexity (tc; depth of interactions between factors of each tree). This approach required investigation of the bag fraction term (bg) that controls overfitting via the introduction of stochasticity to the models (Friedman, 2002). Model parameters were chosen while considering the goodness of fit, as determined via cross‐validation (CV, Table S2). Finally, we used the dependence plots resulted from the BRT models to understand the shape of the influence of every variable in every species.

#### 2.3.3 Predictions of seabird distribution

Spatial predictions in unsampled areas were limited to the convex hull defined within the BIOT MPA and restricted by the range values of the variables used to build each model and the max recorded value of distance from coast (~137 km). This constraint ensured that predictions were only made in areas with similar environmental conditions (see Figure S1 and Table S1). Using this approach, we avoided extrapolating beyond the range of the model, while generating meaningful predictions beyond our sampled area (Yates et al., 2018). Whenever ONI or DMI was retained in the model, we rendered predictions based on the values for the last year of sampling, 2016. We rendered predictions on a 0.4 × 0.4 decimal degree resolved grid. This resolution was considered a reasonable trade‐off in order to capture distribution for species with uncertain range sizes (Seo, Thorne, Hannah, & Thuiller, 2008).

### 2.4 Modeling the effect of rat invasion

We hypothesize that the presence of rat‐invaded islands will influence the distribution of seabirds at sea. We modeled the effect of rat invasion on seabird distribution at sea by modifying our BRTs. We first exchanged the variable distance to coast from each transect with either the distance to the closest rat‐free island or the closest rat‐invaded island. This resulted in two additional models. The model that included “distance to the closest rat‐free islands (km)” was considered to represent bird distribution at its theorized maximum abundance, in the absence of any rat invasion. The model that included “distance to the closest rat‐invaded islands (km)” was considering to represent bird distribution assuming total invasion.

These two models were then used to identify thresholds based on a broken‐line regression analysis of the effect of rat invasion or absence. This analysis gave us the chance to quantify the degree to which seabird distribution is influenced by the distance to rat‐free or rat‐invaded islands using a Davies' test (Davies, 2002). Davies' test enabled us to find the inflexion point of the partial dependence plots by testing the difference in the slopes. The test was done using the R package *segmented* (Muggeo, 2008). This analysis has been previously used to find thresholds on the response to explanatory variables (e.g., Clausen, Christensen, Gundersen, & Madsen, 2017; D'agata et al., 2014; Isles, Xu, Stockwell, & Schroth, 2017; Picard, Rutishauser, Ploton, Ngomanda, & Henry, 2015). As a result, the test provided with break points (BP) of the dependence plots with a range of the 95% confidence intervals (CI). We contrasted the BP and the CI of the rat‐free model, the rat‐invaded model, and the original distribution model.

The final objective of our analysis was to identify possible distribution shifts, after a rat‐eradication scenario. In order to determine the potential net gain in distribution following a scenario of an archipelago‐wide rat‐eradication program, we subtracted the predictions of the rat‐invaded models from the predictions of the rat‐free models. The predictions were mapped only where the nearest island was rat‐invaded since we assume that no new islands will be invaded, showing net gain and net loss in seabird abundance and habitat suitability. An eradication program will not increase a seabird population immediately, as islands may first need to be recolonized, and only after several years of high reproductive output can substantial population increases be expected (e.g., Jones, 2010). Depending on whether an island is actually occupied by a species or not, the initial recolonization may also spill over the abundance elsewhere (as the colonizing birds must come from somewhere). As such, these predictions should be considered as potential only.

## 3 RESULTS

### 3.1 Seabird sightings

In total, 7,008 seabirds were observed during the five expeditions (Table 2). Seven families were recorded: Laridae (noddies and terns), Sulidae (boobies), Procellariidae (shearwaters and petrels), Phaethontidae (tropicbirds), Fregatidae (frigatebirds), Hydrobatidae (northern storm‐petrels), and Oceanitidae (southern storm‐petrels). The most abundant species were red‐footed booby (*Sula sula*: 1,712 individuals and 255 observations, Figure 3a and 3b), brown noddy (*Anous stolidus*: 3,027 individuals and 171 observations, Figure 3c and 3d), white tern (*Gygis alba*: 546 individuals and 154 observations, Figure 3e and 3f), and wedge‐tailed shearwater (*Ardenna pacifica:* 562 individuals and 113 observations, Figure 3g and h). These species were retained for further distribution modeling (Figure 4).

**Figure 1:**
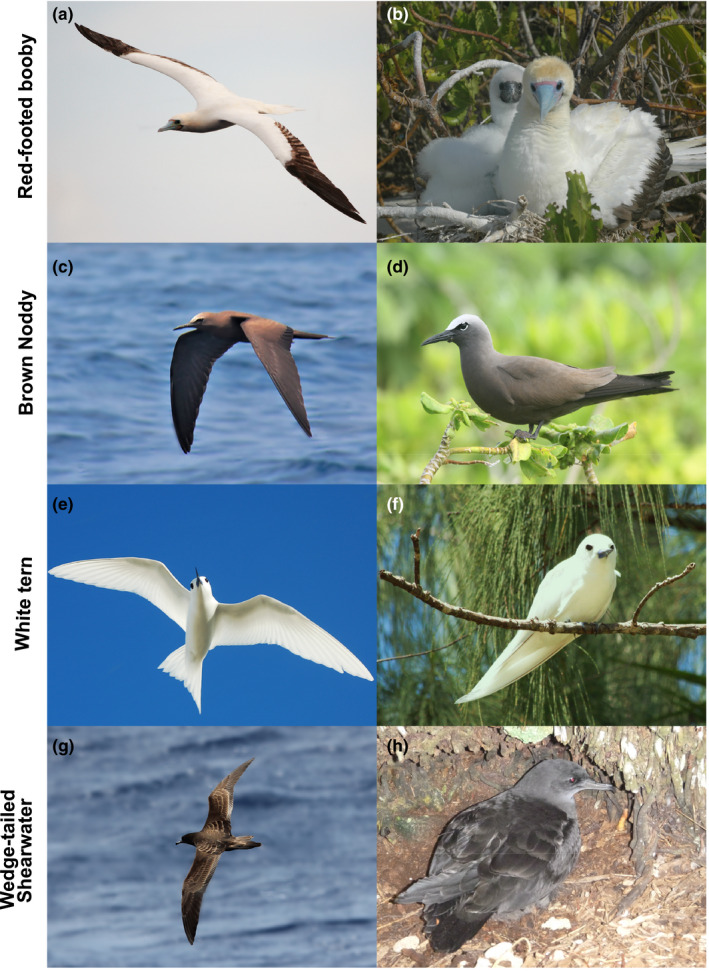
Four most abundant seabird species at sea in the Chagos Archipelago, recorded in flight (a, c, e, and g) and on island for breeding (b and h), or for roosting (d and f). These species were retained for distribution modelling only

**TABLE 2:** Number of observations and individual counts during the survey (2012–2016), separated by family and species

Common Name	Scientific Name	Observations	Individuals count
Laridae (Terns and Noddies)			
Brown Noddy^a^	*Anous stolidus*	171	3,027
Lesser Noddy	*Anous tenuirostris*	41	393
White Tern^a^	*Gygis alba*	154	546
Greater Crested Tern	*Thalasseus bergii*	42	163
Common Tern	*Sterna hirundo*	3	3
Black‐naped Tern	*Sterna sumatrana*	9	22
Little Tern	*Sternula albifrons*	1	1
Bridled Tern	*Onychoprion anaethetus*	26	51
Sooty Tern	*Onychoprion fuscatus*	47	179
Sulidae (Boobies)			
Red‐footed Booby^a^	*Sula sula*	255	1,712
Brown Booby	*Sula leucogaster*	36	104
Masked Booby	*Sula dactylatra*	4	5
Procellariidae (Shearwaters and Petrels)			
Wedge‐tailed Shearwater^a^	*Ardenna pacifica*	113	562
Audubon's Shearwater	*Puffinus lherminieri*	25	58
Tahiti petrel	*Pseudobulweria rostrata*	1	1
Bulwer's Petrel	*Bulweria bulwerii*	18	26
Jouanin's Petrel	*Bulweria fallax*	2	2
Fregatidae (Frigatebirds)			
Great Frigatebird	*Fregata minor*	39	104
Lesser Frigatebird	*Fregata ariel*	6	17
Hydrobatidae (Boreal Storm‐petrels)			
Wilson's storm‐petrel	*Oceanites oceanicus*	1	2
Matsudaira's storm‐petrel	*Oceanodroma matsudairae*	6	22
Phaethontidae (Tropicbirds)			
Red‐tailed Tropicbird	*Phaethon rubricauda*	1	1
White‐tailed Tropicbird	*Phaethon lepturus*	3	6
Oceanitidae (Austral Storm‐Petrels)			
White‐faced Storm‐Petrel	*Pelagodroma marina*	1	1

^a^ Represent the species with the greatest counts used for modeling.

**FIGURE 4:**
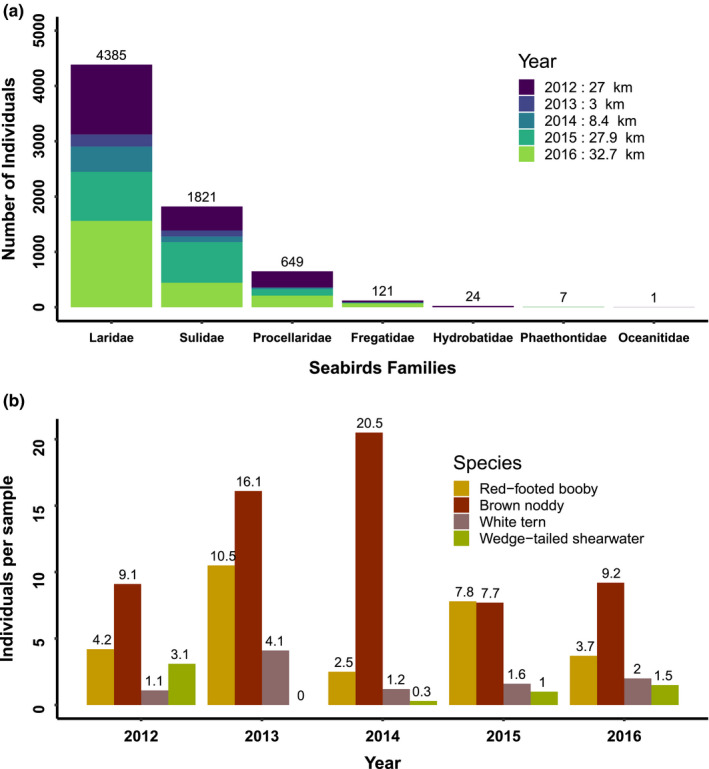
Number of individual seabirds by family summed across year (a) and for the four most abundant species red‐footed booby (*Sula sula*), brown noddy (*Anous stolidus*), white tern (*Gygis alba*), and wedge‐tailed shearwater (*Ardenna pacifica*) standardized by the number of samples for each year (b)

### 3.2 Predictive modeling

#### 3.2.1 Oceanic drivers of distribution and spatial patterns

**FIGURE 5:**
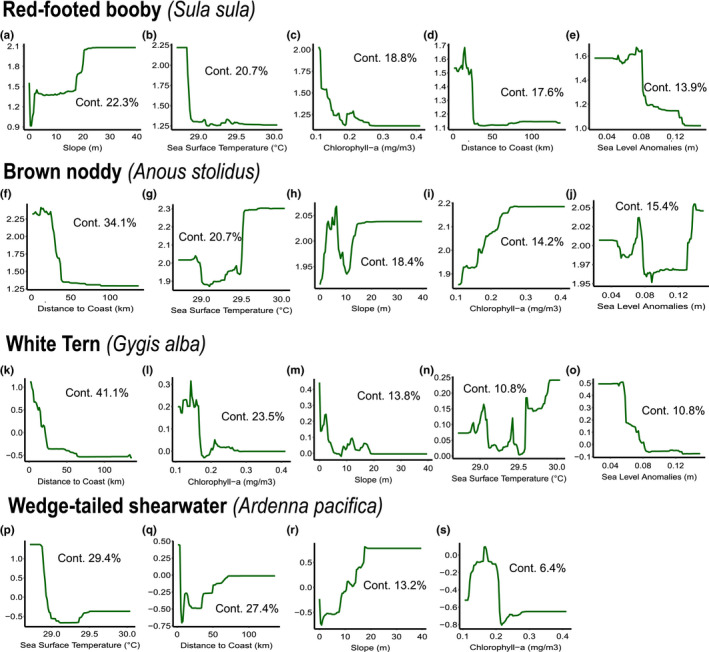
Partial dependence plots of each variable modeled in the BRTs for all four species red‐footed booby (a–e), brown noddy (f – g), white tern (k – o) and wedge–tailed shearwater (p – s). The green solid line in each graph represents the response of the species to the variables. The relative contribution of the model is showed from the greatest to the least. Plots manifest that nonmonotonic responses were found

**FIGURE 6:**
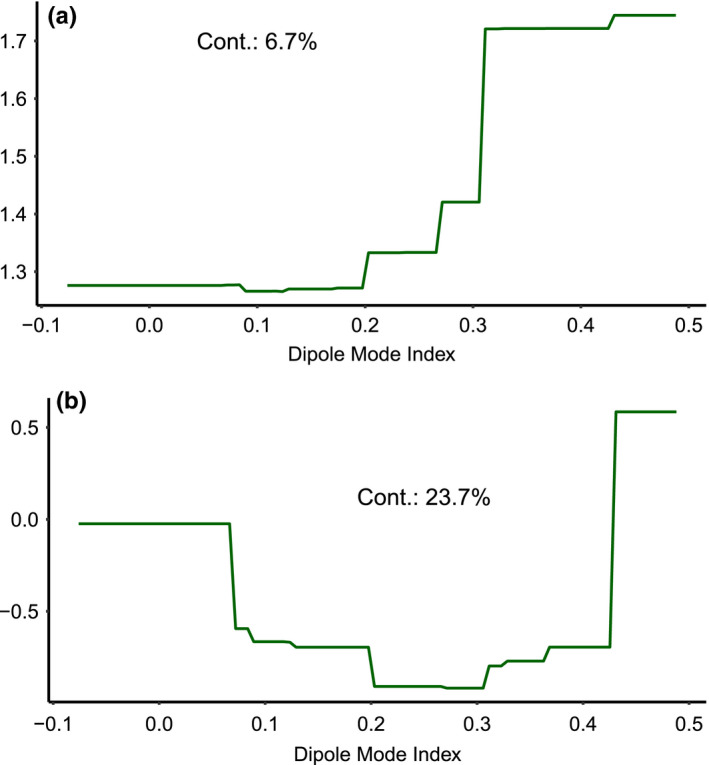
Partial dependence plots of the response of red‐footed booby (a) and wedge‐tailed shearwater (b) to Dipole Mode Index. Both species show a positive correlation with increasing positive DMI. Positive DMI means cooler temperature in eastern Indian Ocean and warmer temperature in western Indian Ocean

Total deviance explained for each BRT was 80% for red‐footed booby, 89% for brown noddy, 88% for white tern, and 99% for wedge‐tailed shearwater. Distance to coast was an important variable for all species, explaining between 17.6% and 41.1% of the deviance (Figure 5b, 5g, 5k and 5q). Slope was a particularly important variable influencing for red‐footed booby (22.3% contribution, Figure 4a) whereas sea surface temperature was the most important for wedge‐tailed shearwater (29.4%). Chlorophyll a concentration explained between 6.4% and 23.5% for all species. DMI was retained for red‐footed booby (6.7%, Figure 6a) and wedge‐tailed shearwater (23.7%, Figure 6b), with both species showing increasing abundance with positive values of the DMI.

**FIGURE 7:**
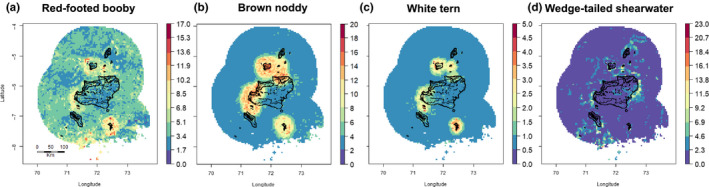
Predicted distribution of red‐footed booby, brown noddy, white tern, and wedge‐tailed shearwater as a function the BRT models. Color scales represent the seabird abundance response, ranging from highest predicted value (red) to zero (blue). Scales differ between species as a function of difference in abundance. Predictions were run within the convex hull of the variables

Spatial predictions for red‐footed booby, brown noddy, and white tern revealed a strong coast signature (Figure 7a–c), while wedge‐tailed shearwater distribution was more uniform with higher abundance levels near high slope areas and toward the northeast of the Archipelago (Figure 7d). Brown noddy and white tern abundance was pronounced over shallow seabeds (<1,000 m) in proximity to islands and atolls (Figure 7b,c). Red‐footed booby abundance was more pronounced in pelagic and deeper areas and in areas with intermediate slope (ca. 15º, Figure 7a).

#### 3.2.2 Response to rat invasion

**FIGURE 8:**
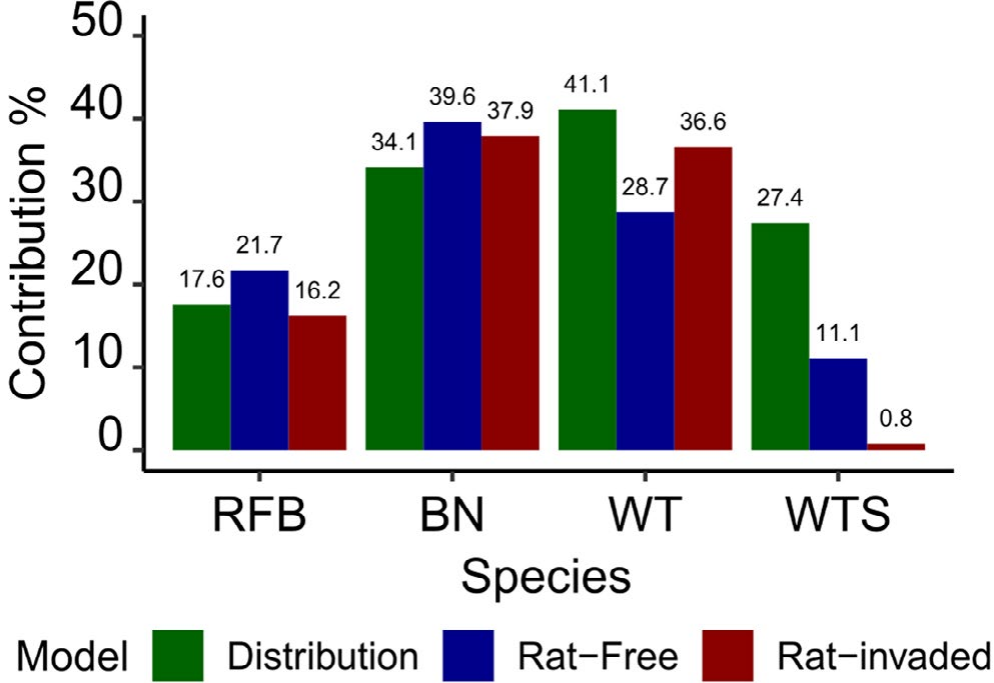
Contribution of the distance to coast on the distribution model, rat‐free model, and rat‐invaded model for red‐footed booby (RFT), brown noddy (BN), white tern (WT), and wedge‐tailed shearwater (WTS). Contribution of distance to a near rat‐free island was greater in red‐footed booby and brown noddy. White tern showed that rat‐invaded island has a greater contribution. Wedge‐tailed shearwater showed difference in contribution between rat‐free and rat‐invaded island but the contribution of distance to coast in the distribution model was greater

The rat‐invaded BRT models outperformed the rat‐free models for the red‐footed booby (86% vs. 82%, total deviance explained), the brown noddy (90% vs. 85%), and the wedge‐tailed (97% vs. 94%). The contribution of distance to rat‐free island was higher than distance to rat‐invaded island for red‐footed booby (21.7% vs. 16.2%) and wedge‐tailed shearwater (11.1% vs. 0.8%) and to a lesser degree for brown noddy (39.6% vs. 37.9%) Conversely, for white tern, distance to rat‐free island explained less deviance (28.7%) than distance to rat‐invaded island (36.6%; Figure 8).

**FIGURE 9:**
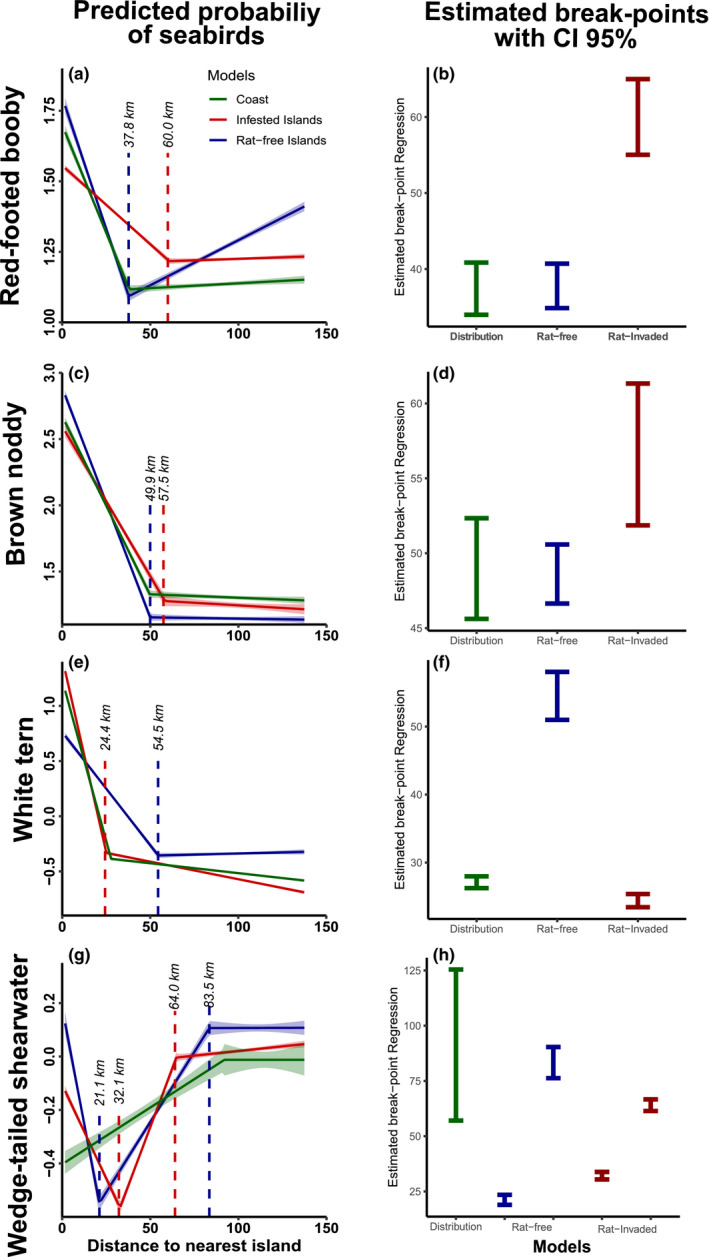
Sensitivity of seabird distribution to presence or absence of rats on nearby islands. Partial dependence plot (a, c, e, g) of the effects of rat‐free (blue lines) and rat‐invaded (red lines) islands contrasted with the original distribution model (green lines). Stippled line denotes location of the breaking point that denotes a threshold in distribution. Comparison of the break point regression using the 95% confident intervals calculated in Davies’ test is shown (b, d, f, h)

Breaking points (BP) indicated the threshold to which the nearest island, whether invaded or not, influenced the distribution of seabirds. Thresholds BP in the effect of islands differ among all species (Figure 9). The BP for red‐footed booby was 37.8 km for rat‐free model [CI 39.4, 40.7] and at 60.0 km for rat‐invaded model [CI 55.2, 65.0]. The BP for brown noddy was at 48.6 km [CI 46.6, 50.6] for rat‐free model and 56.6 km for rat‐invaded model [CI 51.9, 61.3]. The BP for white tern was 54.5 km for rat‐free model [CI 50.9, 58.0] and 24.4 km [CI 23.4, 25.4] for rat‐invaded model. The effect on wedge‐tailed shearwater showed a bimodality with different response from lesser and greater distance. At lesser distance, the BP was 21.2 km for rat‐free model [CI 18.9, 23.5] and 32.1 km [CI 30.4, 33.8] for rat‐invaded model (Table 3).

**TABLE 3:** Results of the broken‐stick regression for the boosted regression trees models considering the original distribution model, a rat‐free model (where we exchanged the variable distance to coast with distant to the nearest ret‐free island) and the rat‐invaded model (where we replaced distance to coast by distance to nearest rat‐invaded island)

Species	Models	Estimated break point (km)	Confidence Intervals (km)	
			Lower	Upper
Red‐footed booby	Distribution Model	37.4	34.0	40.8
	Rat‐invaded Model	60.0	55.0	65.0
	Rat‐free Model	37.8	34.9	40.7
Brown noddy	Distribution Model	49.0	45.6	52.3
	Rat‐invaded Model	56.6	51.9	61.3
	Rat‐free Model	48.6	46.6	50.6
White tern	Distribution Model	27.1	26.2	27.9
	Rat‐invaded Model	24.4	23.4	25.4
	Rat‐free Model	54.5	50.9	58.0
Wedge‐tailed shearwater	Distribution Model	91.2	57.1	125.4
	Rat‐invaded Model	32.1	30.4	33.8
		64.0	61.4	66.7
	Rat‐free Model	21.2	18.9	23.5
		83.3	76.3	90.3

Note

The broken‐stick regression is represented by the breaking point which is the inflection point of the partial dependence plot from each model. Lower and upper confidence interval at 95% is reported in each break point.

**FIGURE 10:**
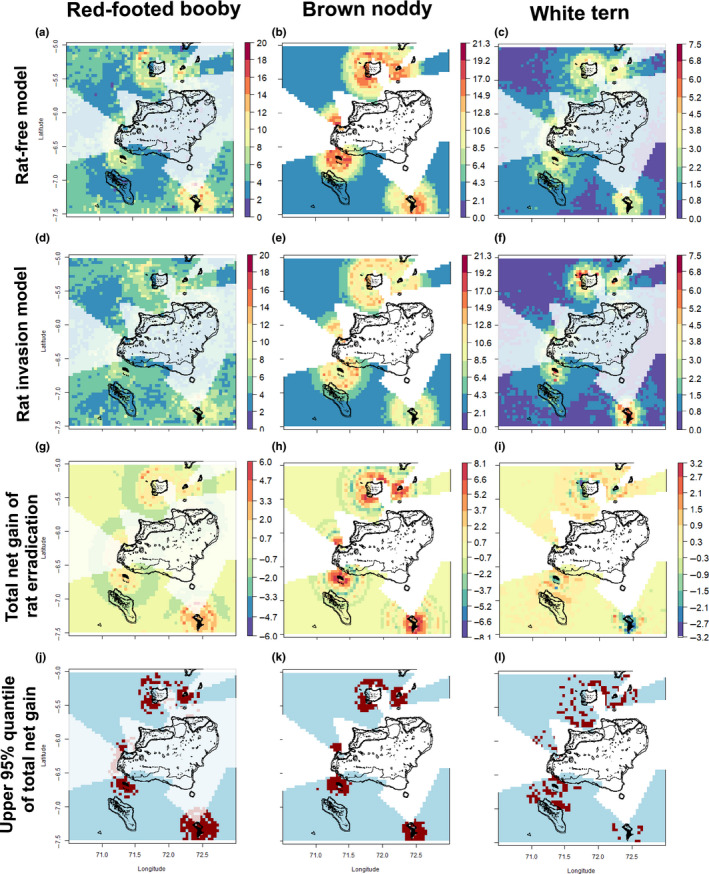
Predicted areas of increase in seabird abundance under a scenario of rat eradication. Rat‐free distribution predicted from BRT model using a rat‐free scenario (a–c), and a rat‐invaded scenario (d–f), where each island is set as either “rat‐free” or “rat‐invaded,” respectively. These models represent the distribution predictions under complete eradications of rats and a full invasion of rats, respectively. Hence, an approach to understand the variations of distribution is calculated by subtracting the rat‐invaded minus the rat‐free models. This yielded to the net gain in bird abundance under a scenario of rat eradication (g–i). The upper 95% quantile increase of the net gain is also showed (j–l). White shaded areas indicate areas nearby currently rat‐free islands in which a rat‐eradication program would not have any effect since they are already at the theorized maximum abundance

The presence of rats on nearby islands reduced the suitable habitat of seabirds (Figure 10). Following rat eradication, abundance at sea of red‐footed booby, brown noddy, white tern, and wedge‐tailed shearwater increased by 14%, 17%, 3%, and 4% respectively. However, the models for wedge‐tailed shearwater distribution were weak and the effect was negligent. Hence, it is not reported in figure.

## 4 DISCUSSION

Using our at‐sea observations in the Chagos Archipelago over a 5‐year period, we have identified spatiotemporal trends in seabird distribution. Our multiyear survey spanned a broad range of environmental conditions and has enabled us to identify how geomorphic and oceanographic variability drives seabird distribution, including a possible association between regional abundance and the IO Dipole oscillation through the DMI. Furthermore, we have modeled the spatially explicit impact of islands with and without rats on seabird distribution and have identified areas of net increase in abundance under an archipelago‐wide rat‐eradication scenario, adding to a mounting body of research on the considerations for rat‐eradication priorities, both globally (Buxton, Jones, Moller, & Towns, 2014) and in BIOT (Dawson et al., 2015). Information on seabird hotspots, sensitivity to climate oscillation, and how eradication can result in distribution shifts has critical implications for tropical seabird conservation and for island restoration strategies.

### 4.1 Drivers of seabird distribution within the Chagos Archipelago

The red‐footed booby showed a primarily oceanic distribution, with pronounced hotspots east of Diego Garcia, northwest of Peros Banhos, and south of Salomon. Red‐footed booby distribution has traditionally been thought to be positively associated with areas of high productivity and elevated chlorophyll a concentration (>0.16 mg m^−3^ chl‐a) (Ballance, Pitman, & Reilly, 1997; Jaquemet, Le Corre, Marsac, Potier, & Weimerskirch, 2005; Weimerskirch et al., 2005). In contrast, Mendez et al. (2017) identified a negative correlation between red‐footed boobies and chlorophyll a, by tracking foraging behavior of red‐footed boobies in colonies along the equator (Galapagos Islands, Mozambique Channel, New Caledonia, and the IO) which permitted a more robust understanding of factors that determines distribution. Mendez et al. (2017) concluded that across the pantropical range of the red‐footed booby, distribution is closely driven by intra‐ and interspecific competition for prey. As chlorophyll a concentration was negatively correlated with red‐footed boobies here, our results appear consistent with Mendez et al. (2017), with competition being an important driver of distribution. Red‐footed booby were sensitive to rats, aggregating in greater abundance near rat‐free islands.

Brown noddy and white tern distributions were strongly related to distance to coast, concentrating around islands, and over shallow water (<1,000 m). Furthermore, brown noddy distribution was associated with high sea surface temperature (>29.5°C), while white terns were strongly influenced by low chlorophyll a concentration (<0.2 mg m^−3^ chl‐a). Our results are in line with previous observations of brown noddies leaving and returning to islands during the same day in the breeding season, suggesting they are not long‐distance or multiday foragers (Jaquemet, Le Corre, & Weimerskirch, 2004; Surman, Nicholson, & Ayling, 2017). The two species appeared to have different responses to rat invasion. The reasons for this difference in response remain unknown, but could be linked to the fact that both species breed on rat‐invaded islands (Carr, pers. obs.), thus confounding any signal related to invasion status.

Wedge‐tailed shearwater were restricted to less productive waters (<0.20 mg m^−3^ chl‐a) with high slope (> 20°) and were more attracted to colder water (<29°C) across the BIOT MPA. Our observations were consistent with those of Mannocci, Laran, et al. (2014) in the western IO, reporting lower numbers in productive areas (>0.37 mg/m^3^). The wedge‐tailed shearwater were the least sensitive species to distance to coast and in contrast to the other species appeared to increase in abundance with increasing distance. A potential reason for this is that shearwaters typically have a wider foraging range from nesting colonies (ca. 480 km; King, 1974) than red‐footed booby (ca. 67.5 km; Young et al., 2010) and brown noddy (ca. 80 km; Harrison & Stone‐Burner, 1981; King, 1974). Therefore, this response is likely an artifact of the observed birds commuting to foraging areas, rather than actually foraging in the relatively near‐coast areas. Wedge‐tailed shearwater return to burrows only at night, so that their distribution appears independent from islands may be due to a predominantly scattered and remote distribution during the day (Dias, Alho, Granadeiro, & Catry, 2015). Although we found no significant effect of rat invasion on wedge‐tailed shearwater distribution, many shearwater species and allies are particularly vulnerable to invasive land predators (Dias et al., 2019; Smith, Polhemus, & VanderWerf, 2002), because they nest in ground burrows. The pelagic behavior and large foraging range which our sampling range failed to capture may mask any distribution shift related to rat invasion and the other environmental variables, as indicated by the weaker models.

Constant competition over prey is expected to lead to a prey depredation zones around colonies, otherwise known as Ashmole's Halo (Ashmole, 1963). Halos vary as a function of colony, size, and bird foraging range (Birt, Birt, Goulet, Cairns, & Montevecchi, 1987). Within the Chagos Archipelago, many islands are <100 km apart and are clustered close together (<20 km between islands) within atolls. The range to which the abundant red‐footed booby, brown noddy, and white tern distributions radiate out from islands (i.e., 263, 136, and 133 km respectively) makes it therefore very likely that neighboring colonies compete, either by overlapping in distribution or by expressing behavior to minimize foraging overlap (Mendez et al., 2017; Wakefield et al., 2013). The wider distribution range and lower abundance of the wedge‐tailed shearwater make competition between colonies less likely than for other seabirds (Gaston, Ydenberg, & Smith, 2007).

### 4.2 Influence of climate oscillation

Our multiyear time series enabled us to document effect of climatic oscillations at the interannual scale on distribution. We observed similar abundance trends for red‐footed booby and wedge‐tailed shearwater during the 5 years of sampling, with both species abundance positively correlating with the DMI. We detected no correspondence between seabird abundance and the ONI, a proxy for the ENSO index. These observations are consistent with current understanding regarding the influence of the dipole of higher trophic levels in the Indian Ocean, adding to a limited but growing body of research on the importance of the Dipole on IO megafauna. For example, Kumar, Pillai, and Manjusha (2014) identified a positive association between IO tuna productivity and the Dipole Index. In the southern IO, albatross breeding success has also been positively correlated with the Dipole Index (Rivaland et al., 2010). The knowledge of mechanisms driving these patterns is at present limited and any explanation must remain speculative at this stage. There is evidence that equatorial upwellings in the IO are more pronounced and that westerly winds decrease in intensity during positive Dipole events (Du & Zhang, 2015). In the Chagos Archipelago, it is thus possible that negative Dipole events result in weakened regional upwelling and therefore require seabirds to forage further afield, leading to a drop in regional abundance. This would be consistent with present understanding regarding other climate oscillations such as ENSO, which is known to influence forage species productivity (Lehodey, Bertignac, Hampton, Lewis, & Picaut, 1997), with implications for higher trophic levels. For example, common bottlenose dolphin (*Tursiuops trunctatus*) migrate offshore during strong ENSO years, possibly due to a lack of inshore prey (Sprogis et al., 2018). Use of telemetry and satellite tracking is currently being deployed on red‐footed boobies in BIOT (Carr, pers. obs.), which will enable mechanisms to be explored in more detail. The greater sensitivity of wedge‐tailed shearwater to both oceanographic variables and to the Dipole suggests this family may be the most vulnerable to global environmental change.

Any linkage between the Dipole and mobile megafauna is likely mediated by multiple trophic links (Oro, 2014). As foragers commensal with subsurface predators, seabirds could be impacted by the Dipole both directly, for example, by a reduction in forage species abundance, and indirectly, by an increase in tuna abundance (Kumar et al., 2014; Maxwell & Morgan, 2013). It is beyond our scope to distinguish these processes here; however, we are currently expanding our analysis of seabird distribution to include data on subsurface prey and predator abundance collected simultaneously to the seabird observations, using midwater baited videography (Letessier, Bouchet, & Meeuwig, 2017; Letessier et al., 2019).

### 4.3 Implication for rat‐eradication programs

Past rat‐eradication efforts in the Chagos Archipelago include a failed attempt on Eagle Island (Meier, 2006) and successful attempts on Îles Vache Marine, du Sel, and Jacobin (Harper et al., 2019). The latter attempts were focussed on small islands to test the feasibility of eradication and appropriate methodologies on a small scale. Island rodent eradication is increasingly recognized as a powerful strategy for the preservation and recovery of avian populations (Brooke et al., 2018; Jones et al., 2016; Lavers, Wilcox, & Donlan, 2010). However, eradication is technically challenging and expensive (Warren, 2018), requiring the application of toxic rodenticide posing a risk to humans, livestock, pets, and wildlife (Pickrell, 2019; Van den Brink, Elliott, Shore, Rattner, 2018). Eradication is more likely to fail in the tropics, with high mean annual temperatures and constant precipitation (Russell & Holmes, 2015), and in the presence of land crabs and coconut palms (Holmes et al., 2015), making a program in the Chagos Archipelago challenging. Eradication on the largest island of Diego Garcia is likely to be particularly complex and expensive as it is inhabited (Harper & Carr, 2015).

Our analysis has revealed potential increases in habitat usage following rat eradication and that these habitats are spatially and species‐specific. On the basis of our study, we propose that eradication should be prioritized on Île Manoel, Île Yeye, and Île de la Passe in Peros Banhos Atoll and on Eagle Island in the western Great Chagos Bank. In addition to minimizing overlap between distributions of the recovering colonies, these islands fulfill all the criteria identified by Buxton et al. (2014), such as proximity to healthy metapopulation and seabird diversity. Our recommendations are consistent with those of Dawson et al. (2015), which rank Île de la Passe in the top 25 islands for invasive vertebrate eradication in the UK overseas territories. We note that these recommendations are on the basis of factors explored in this study only and that there are other factors that dictate the feasibility, success, and approach to rodent eradication. Our results here aim to form part of a far wider set of considerations, with the ultimate aim of eradicating rats from all islands in the archipelago, in order to achieve full conservation impact.

### 4.4 Concluding remarks

Seabird abundance and distribution at sea in BIOT are driven by geomorphology and oceanographic conditions. Our distribution predictions complement previous efforts elsewhere in the IO, and our time series has enabled us to identify potential interannual variability related to climate oscillation. Seabird populations are vulnerable to both climatic variability and human activities (Dias et al., 2019; Paleczny et al., 2015). Environmental variability is predicted to increase globally under climate change scenarios (IPCC, 2014), and evidence suggests that global warming variability may decouple the Dipole from upwelling in the western IO (Watanabe, Watanabe, Yamazaki, Pfeiffer, & Claereboudt, 2019). Identifying how interannual processes like the IO Dipole drives seabird distribution where human activities are limited is valuable for identifying long‐term strategies for seabird protection. For example, our predictions will enable responses to predicted extreme climatic event to be anticipated and thus mitigated in spatial management regimes.

To our knowledge, this is the first attempt at predicting the potential response of seabird distribution by predicting potential shifts in habitats usage following a rat‐eradication scenario. We have demonstrated areas of potential distribution gain and have predicted new hotspots at sea following a rat‐eradication program. There is considerable impetus for eradicating invasive species on islands (Brooke et al., 2018; Dawson et al., 2015; Holmes et al., 2019; Jones et al., 2016; Lavers et al., 2010), further supported by our research here and other related research in the Chagos Archipelago (Benkwitt et al., 2019, 2020; Graham et al., 2018; Harper et al., 2019). In addition to practical considerations such as cost and probability of success, eradication programs should identify where eradication can have the greatest conservation potential and ecological impact. This is particularly important for seabirds, whose niche extends beyond terrestrial breeding colonies.

## CONFLICT OF INTEREST

None declared.

## AUTHOR CONTRIBUTIONS

**Julian Perez‐Correa:** Conceptualization (lead); formal analysis (lead); methodology (lead); visualization (lead); writing – original draft (lead); writing – review and editing (lead). **Peter Carr:** Investigation (lead); methodology (equal); Supervision (supporting); validation (equal); writing – review and editing (supporting). **Jessica J. Meeuwig:** Conceptualization (supporting); investigation (supporting); methodology (supporting); supervision (supporting); validation (supporting); visualization (supporting); writing – original draft (supporting); writing – review and editing (supporting). **Heather J. Koldewey:** Supervision (supporting); validation (supporting); writing – original draft (supporting); writing – review and editing (supporting). **Tom B. Letessier:** Conceptualization (equal); formal analysis (lead); investigation (supporting); methodology (supporting); validation (equal); writing – original draft (lead); writing – review and editing (lead).

### Open Research Badges

This article has earned an Open Materials Badge for making publicly available the components of the research methodology needed to reproduce the reported procedure and analysis. All materials are available at https://github.com/juperez/SeabirdChagos.

## Supporting information

Appendix S1

## Data Availability

Data used in this research are publicly available at https://doi.org/10.5061/dryad.sxksn0311. The R code used to conduct this research is available for public access in https://github.com/juperez/SeabirdChagos.
