# Supplementary material for: Climate oscillation and the invasion of alien species influence the oceanic distribution of seabirds
Source: Ecol Evol. 2020 Aug 20;10(17):9339–57. doi: 10.1002/ece3.6621 (PMC7487247; doi:10.1002/ece3.6621)
Supplement: Supplementary file 1 — Appendix S1 [file ECE3-10-9339-s001.docx]

# APPENDIX S1

# S1. Variables selection

Elevated SLA and SST values were located to the north of the archipelago whereas chlorophyll-a values typically increased in proximity with the atolls (Figure S2 c-e). Dipole Mode Index (DMI) and the Oceanic Niño Index (ONI) were modelled to capture the variability and the potential influence of climate oscillation. The spatial resolution for oceanographic variables was monthly climatological, as recommended by Mannocci et al., 2017 (Table S1).

**TABLE S1.** Oceanographic and geomorphic covariates used to construct and fit seabird models. Resolution of original data is provided

| **Geomorphic variables** | **Units** | **Range** | **Temporal Resolution** | **Spatial Resolution** |
| --- | --- | --- | --- | --- |
| Distance to coast | km | 1.1 to 136.5 | - | 0.83 km |
| Slope (Slope) | % | 0 to 39.6 | - | 0.83 km |
| **Oceanographic variables** | **Units** | **Range** |  |  |
| Chlorophyll-a concentration (CHL) | mg/m^3^) | 0.1 to 0.41 | Monthly Climatology | 4 km |
| Sea surface temperature (SST) | C° | 28.7 to 30.04 | Monthly Climatology | 4 km |
| Sea-levels anomalies (SLA) | m | 0.03 to 0.015 | Daily | 24 km |
| **Climatic variability variables** | **Units** | **Range** |  |  |
| Year | - | 2012 to 2016 | Yearly | - |
| Dipole Mode Index | - | -0.076 to 0.488 | Monthly | - |
| Oceanic Niño Index | - | -0.3 to 2.2 | Montly | - |

*Note.* Selected covariates were chosen considering other research done in seabirds and top-marine predators by Fox et al. (2017); Mannocci, Catalogna, et al. (2014); Mannocci, Laran, et al. (2014); Vilchis et al. (2006).

**
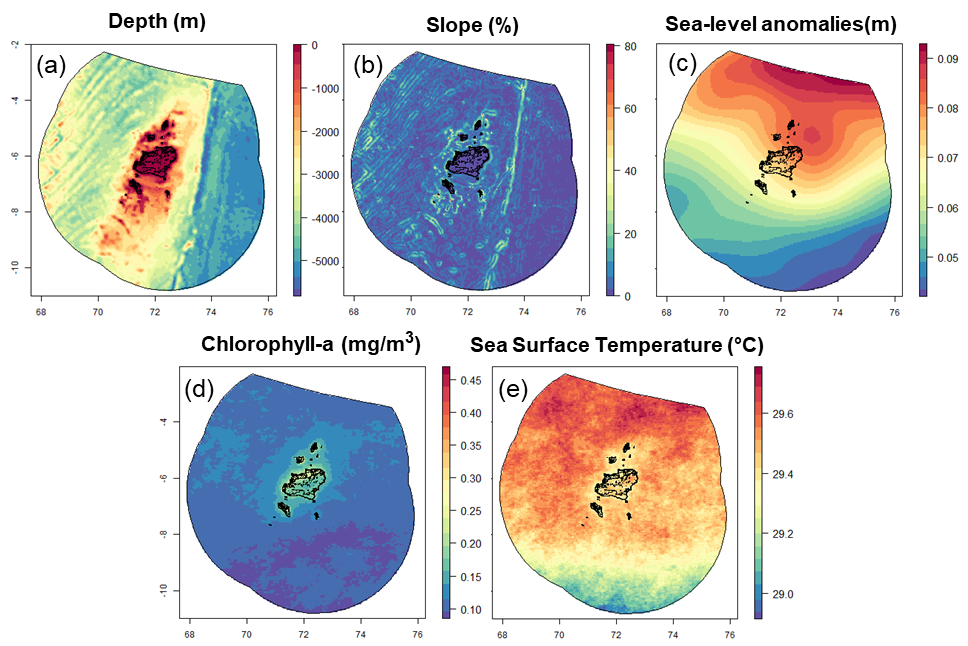
**

**FIGURE S1.** Geomorphic and oceanographic covariates used in modelling. Time-average values are shown for Sea Levels Anomalies (c), Chlorophyll-a concentration (d) and, Sea Surface Temperature (d).

# S3. Boosted Regression Tree

Non-linear relationships are common in nature. However, identifying thresholds are usually problematic, unless one assumes linearity with specific breaking points (Potts & Elith, 2006; Folke et al., 2004; Elmqvist et al., 2003). Rats are known to significantly impact seabirds by predating on eggs and harassing breeding colonies (King, 1985), although it is unclear if rat presence on nearby island leads to any distribution shift in seabirds at sea, beyond the immediate terrestrial decline. Previous research on seabird suggests that rat effects on seabird are taxa-specific, with vulnerability in part related to breeding and nesting behaviour (Jones et al., 2008). Using a boosted regression tree (BRT) model we explored non-monotonic relationships of distance from coast to rat-free islands or rat-infested island. We used the package gbm on R with recommendations by Elith et al., (2008). As they suggested we did an analytical exploration of BRT that lead to specific model parameters (Table S2).

**TABLE S2.** Boosted Regression Tree parameters used to fit the models on specific family.

| **Model features** | **Red-footed booby** | **Brown noddy** | **White tern** | **Wedge-tailed shearwater** |
| --- | --- | --- | --- | --- |
| **Tree complexity** | 4 | 4 | 4 | 1 |
| **Numbers of trees** | 1000 | 1000 | 1000 | 1000 |
| **Learning rate** | 0.0001 | 0.0001 | 0.0001 | 0.0001 |
| **Bag fraction** | 0.5 | 0.5 | 0.5 | 0.3 |

We applied a large numbers of trees (1000) and a low learning rate (0.0001). This combination reduced the stochasticity of computation, because it decreases the influence of the subsequent trees formed in the boosting process (Elith et al., 2008).

**TABLE S3.** Boosted Regression Tree results from the models. We report the deviance explained by each model, as well as the cross validation scores and the respective standard error as representation of the uncertainty of the models.

| **Specie** | **Type of model** | **Null Deviance** | **Residual Deviance** | **Deviance Standard Error** | **Cros Validation Score** | **Correlation Standard Error** | **Total Deviance Explained** |
| --- | --- | --- | --- | --- | --- | --- | --- |
| **Red-footed booby** | **Distribution Model** | 8.5 | 6.8 | 1.3 | 0.4 | 0.05 | 0.80 |
|  | **Rat-invaded model** | 8.5 | 7.3 | 1.2 | 0.4 | 0.07 | 0.86 |
|  | **Rat-free model** | 8.5 | 6.9 | 0.7 | 0.4 | 0.05 | 0.82 |
| **Brown noddy** | **Distribution Model** | 19.1 | 16.8 | 0.7 | 0.3 | 0.05 | 0.88 |
|  | **Rat-invaded model** | 19.1 | 17.2 | 1.4 | 0.3 | 0.05 | 0.90 |
|  | **Rat-free model** | 19.1 | 16.2 | 1.0 | 0.4 | 0.02 | 0.85 |
| **White tern** | **Distribution Model** | 4.2 | 3.7 | 0.6 | 0.3 | 0.08 | 0.88 |
|  | **Rat-invaded model** | 4.2 | 3.5 | 0.5 | 0.4 | 0.07 | 0.84 |
|  | **Rat-free model** | 4.2 | 3.8 | 0.6 | 0.4 | 0.06 | 0.90 |
| **Wedge-tailed shearwater** | **Distribution Model** | 7.6 | 7.5 | 3.7 | 0.2 | 0.05 | 0.99 |
|  | **Rat-invaded model** | 7.6 | 7.4 | 3.7 | 0.2 | 0.05 | 0.97 |
|  | **Rat-free model** | 7.6 | 7.1 | 3.2 | 0.2 | 0.05 | 0.94 |

# S2. Seabird Individual Counts

We opted to conduct our analysis of seabird distribution retaining only species with sufficient observations ( >100), in order to ensure adequate statistical power. The species represented three families Sulidae (red-footed booby, *Sula sula*), Laridae (brown noddy, *Anous stolidus* and white tern *Gygis alba*) and Procellariidae (wedge-tailed shearwater, *Puffinus pacificus*). Sulidae, Laridae and Procellaridae were recorded in all sectors of the Chagos Archipelago. Other families were also recorded in the expeditions. Fregatidae (frigatebirds) were primarily found in the northern sections, near Salomon and Peros Banhos atolls. Hydrobatidae (storm-petrels) were abundant in proximity to the Salomon atoll, The Three Brothers and east of Diego Garcia, and were absent from the Great Chagos Bank. Phaethontidae (tropicbirds) and Oceanitidae (Austral Storm-Petrels) were only found in the northernmost area of the archipelago (Figure S3).


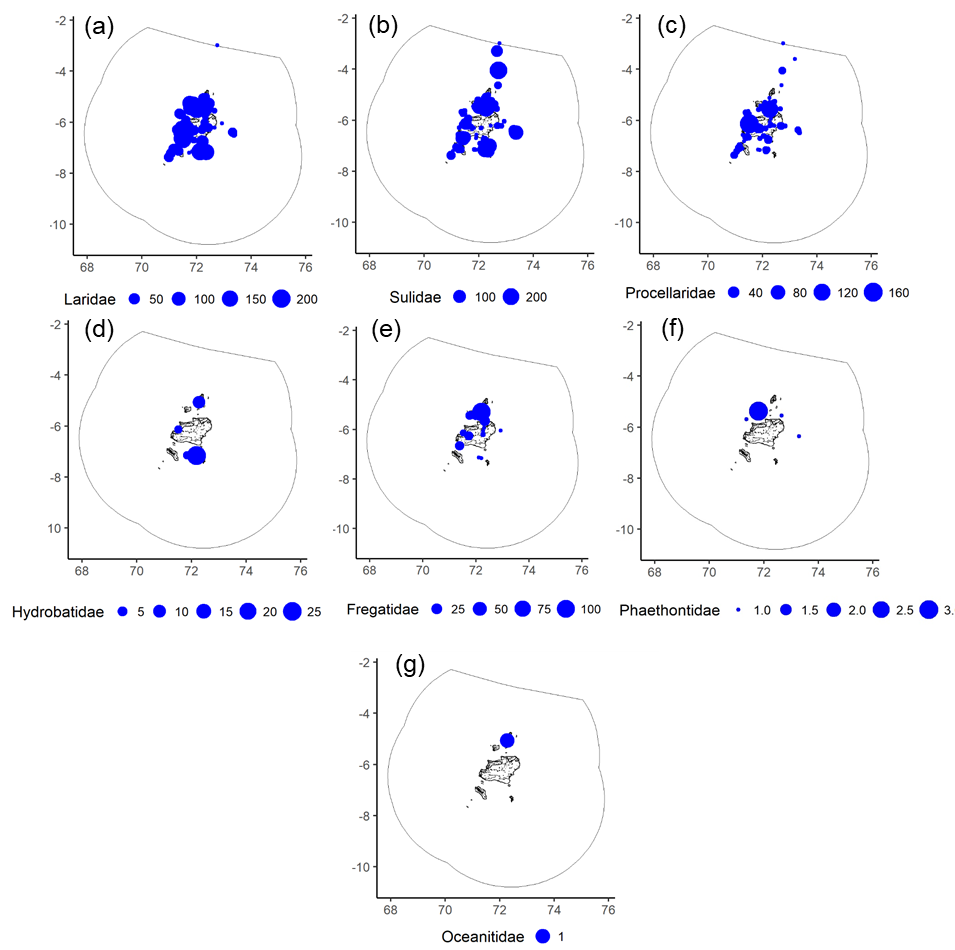


**FIGURE S3**. Seabird observations at sea between 2012 and 2016, around the Chagos Archipelago, grouped by family.

# References

Elith, J., Leathwick, J. R., & Hastie, T. (2008). A working guide to boosted regression trees. *Journal of Animal Ecology, 77*(4), 802-813. doi: 10.1111/j.1365-2656.2008.01390.x

Elmqvist, T., Folke, C., Nyström, M., Peterson, G., Bengtsson, J., Walker, B., & Norberg, J. (2003). Response diversity, ecosystem change, and resilience. *Frontiers in Ecology and the Environment, 1*(9), 488-494. doi: 10.1890/1540-9295(2003)001[0488:RDECAR]2.0.CO;2

Folke, C., Carpenter, S., Walker, B., Scheffer, M., Elmqvist, T., Gunderson, L., & Holling, C. S. (2004). Regime shifts, resilience, and biodiversity in ecosystem management. *Annual Review of Ecology, Evolution, and Systematics, 35*, 557-581. doi: 10.1146/annurev.ecolsys.35.021103.105711

Fox, C. H., Huettmann, F. H., Harvey, G. K. A., Morgan, K. H., Robinson, J., Williams, R., & Paquet, P. C. (2017). Predictions from machine learning ensembles: marine bird distribution and density on Canada’s Pacific coast. *Marine Ecology Progress Series, 566,* 199-216. doi: 10.3354/meps12030

Jones, H. P., Tershy, B. R., Zavaleta, E. S., Croll, D. A., Keitt, B. S., Finkelstein, M. E., & Howald, G. R. (2008). Severity of the effects of invasive rats on seabirds: a global review. *Conservation Biology, 22*(1), 16-26. doi: 10.1111/j.1523-1739.2007.00859.x

King, W. B. (1985). Island birds: will the future repeat the past. *Conservation of island birds, 3*, 3-15.

Mannocci, L., Boustany, A. M., Roberts, J. J., Palacios, D. M., Dunn, D. C., Halpin, P. N., ...Bograd, S. J. (2017). Temporal resolutions in species distribution models of highly mobile marine animals: Recommendations for ecologists and managers*. Diversity and Distributions, 23*(10), 1098-1109. doi: 10.1111/ddi.12609

Mannocci, L., Laran, S., Monestiez, P., Dorémus, G., Van Canneyt, O., Watremez, P., & Ridoux, V. (2014). Predicting top predator habitats in the Southwest Indian Ocean. *Ecography, 37*(3), 261-278. doi: 10.1111/j.1600-0587.2013.00317.x

Maxwell, S. M., & Morgan, L. E. (2013). Foraging of seabirds on pelagic fishes: implications for management of pelagic marine protected areas*. Marine Ecology Progress Series, 481*, 289-303. doi: 10.3354/meps10255

Potts, J. M., & Elith, J. (2006). Comparing species abundance models. *Ecological modelling, 199*(2), 153-163. doi: 10.1016/j.ecolmodel.2006.05.025

Vilchis, L. I., Ballance, L. T., & Fiedler, P. C. (2006). Pelagic habitat of seabirds in the eastern tropical Pacific: effects of foraging ecology on habitat selection. *Marine Ecology Progress Series, 315*, 279-292. doi: doi:10.3354/meps315279
